# Correlation of Quantitative Motor State Assessment Using a Kinetograph and Patient Diaries in Advanced PD: Data from an Observational Study

**DOI:** 10.1371/journal.pone.0161559

**Published:** 2016-08-24

**Authors:** Christiana Ossig, Florin Gandor, Mareike Fauser, Cecile Bosredon, Leonid Churilov, Heinz Reichmann, Malcolm K. Horne, Georg Ebersbach, Alexander Storch

**Affiliations:** 1 Division of Neurodegenerative Diseases, Department of Neurology, Technische Universität Dresden, D-01307, Dresden, Germany; 2 Movement Disorders Clinic, D-14547, Beelitz-Heilstätten, Germany; 3 German Center for Neurodegenerative Diseases (DZNE), D-18147, Rostock, Germany; 4 Florey Institute for Neuroscience and Mental Health, University of Melbourne, Heidelberg, 3084, Australia, and RMIT University, Melbourne, 3000, Australia; 5 Department of Neurology, Technische Universität Dresden, D-01307, Dresden, Germany; 6 Florey Institute for Neuroscience and Mental Health, University of Melbourne, Parkville, 3010, Australia, and St Vincent’s Hospital, Fitzroy, Victoria, 3065, Australia; 7 Department of Neurology, University of Rostock, D-18147, Rostock, Germany; Oslo Universitetssykehus, NORWAY

## Abstract

**Introduction:**

Effective management and development of new treatment strategies for response fluctuations in advanced Parkinson’s disease (PD) largely depends on clinical rating instruments such as the PD home diary. The Parkinson’s kinetigraph (PKG) measures movement accelerations and analyzes the spectral power of the low frequencies of the accelerometer data. New algorithms convert each hour of continuous PKG data into one of the three motor categories used in the PD home diary, namely motor Off state and On state with and without dyskinesia.

**Objective:**

To compare quantitative motor state assessment in fluctuating PD patients using the PKG with motor state ratings from PD home diaries.

**Methods:**

Observational cohort study on 24 in-patients with documented motor fluctuations who completed diaries by rating motor Off, On without dyskinesia, On with dyskinesia, and asleep for every hour for 5 consecutive days. Simultaneously collected PKG data (recorded between 6 am and 10 pm) were analyzed and calibrated to the patient’s individual thresholds for Off and dyskinetic state by novel algorithms classifying the continuous accelerometer data into these motor states for every hour between 6 am and 10 pm.

**Results:**

From a total of 2,040 hours, 1,752 hours (87.4%) were available for analyses from calibrated PKG data (7.5% sleeping time and 5.1% unclassified motor state time were excluded from analyses). Distributions of total motor state hours per day measured by PKG showed moderate-to-strong correlation to those assessed by diaries for the different motor states (Pearson’s correlations coefficients: 0.404–0.658), but inter-rating method agreements on the single-hour-level were only low-to-moderate (Cohen’s κ: 0.215–0.324).

**Conclusion:**

The PKG has been shown to capture motor fluctuations in patients with advanced PD. The limited correlation of hour-to-hour diary and PKG recordings should be addressed in further studies.

## Introduction

Effective management and development of new treatment strategies for response fluctuations in PD largely depends on clinical rating instruments such as the PD home diary [1], Unified PD rating scale (UPDRS) [2] or modified Abnormal Involuntary Movement Scale (mAIMS)[3]. However, clinical ratings have limitations regarding inter-rater variability and continuous monitoring. Patient diaries are self-completed forms reflecting the patient’s perception of his motor state for every hour awake and are frequently used in PD for both clinical practice and as clinical trial endpoints [4]. Estimation of motor complications using diaries heavily relies on their accurate completion and is thus associated with a large recall bias and diary fatigue, particularly in patients with cognitive dysfunction or depression [4, 5]. Consequently, there is a strong need for continuous and objective monitoring of motor performance in PD for both improving therapeutic regimens in routine care and for usage in clinical trials.

Current approaches to objectively monitor motor function such as bradykinesia and dyskinesia in PD comprise of various wearable sensors (accelerometer with or without gyroscopes) placed on different body parts to measure movements [6–12]. Various studies used gait parameters measured by wearable sensor-based devices to estimate bradykinesia and showed strong correlation of gait parameters with bradykinesia [9, 10, 13, 14]. In order to measure dyskinesia in advanced PD, there are various approaches using data from accelerometers and/or gyroscopes fixed to various parts of the body, in most cases while the patient were performing standardised motor tasks and/or voluntary movements. Although the comparison of the various approaches with their different recording modes and technologies is difficult, the accuracies of the approaches when compared to clinical estimation of dyskinesia are very high [6, 7, 11, 15–18].

The Parkinson’s Kinetigraph logger (PKG; Global Kinetics Corporation, Melbourne, Australia) measures movement accelerations of the wrist and analyzes the spectral power of the low frequencies of accelerometer data providing continuous variables—namely the median bradykinesia score (BKS) and dyskinesia score (DKS)–which closely correlate with UPDRS motor score and mAIMS, respectively [7]. In contrast to most other technologies, the PKG records movements over several days during normal daily life. This fact enabled Horne and co-workers to recently introduce a fluctuation score, which distinguishes between fluctuating and non-fluctuating patients with high sensitivity and selectivity and which might be sensitive to treatment effects [19].

We here determine the agreement of data generated by the PKG measuring movement accelerations of the wrist and by the PD home diary to ask whether the objective assessment that correlates with the UPDRS motor score and AIMS could correlate well with diaries. By using new algorithms designed for the present study, the continuous PKG data from each hour arising out of the algorithm already introduced by Griffith and co-workers in 2012 [7] were converted into one of the three motor categories used for the PD home diary, namely motor Off state and On state with and without dyskinesia.

## Materials and Methods

### Study cohort

Subjects fulfilling UK PD Brain Bank criteria [20] with documented motor fluctuations were enrolled into this observational cohort study between January and May 2013 as inpatients at the Department of Neurology at the Technische Universität Dresden and at the Movement Disorders Clinic Beelitz-Heilstätten. Motor fluctuations were classified as documented if motor fluctuations had been documented by a trained neurologist in the patient’s records and/or reported in the UPDRS part IV (performed by a trained neurologist) [2]. Patients were excluded if they had an identifiable cause of parkinsonism or signs for atypical parkinsonism, psychosis, or dementia (Montreal-Cognitive-Assessment ≤26 points) [21] or other relevant conditions interfering with the study protocol. All patients provided written informed consent and the study was approved by responsible institutional review board of the Technische Universität Dresden and received the study number EK388122012.

### Basic assessments

We assessed basic demographic data including type of motor complication, Hoehn-Yahr score [22], UPDRS [2] and Beck’s Depression Inventory (BDI) [23]. The UPDRS motor score (part III) [2] was assessed during the inpatient period in the defined Off state after drug free period and STN-DBS turned off for at least 12 hours, and best possible motor On state. Patients underwent a diary training session for the PD motor diary introduced by Hauser and colleagues with four different motor states (asleep, motor Off, On without dyskinesia, and On with dyskinesia) [24]. During the training session, patients were instructed as to how the different functional states are defined and how the diaries have to be completed (placing a tick mark on a daily diary card every 60 minutes reflecting their predominant status over the prior hour period; for time asleep, the diary was completed upon awakening). Patients then completed diaries on 6 consecutive days (5 days for analysis plus first day for PKG calibration) without a specific reminder function.

### PKG motor state measurement

The PKG measures acceleration of the wrist and was worn between 6 am and 10 pm on the most severely affected side. The new algorithms designed for the present study are using the continuous variables BKS and DKS arising out of the analyzing technique of the spectral power of the low frequencies of accelerometer data developed by Griffith and colleagues in 2012 [7]. These new algorithms convert each hour of the continuous variables BKS/DKS into one of the five categories (asleep, motor Off state, On state or On state with dyskinesia [also: dyskinetic state], not wearing PKG) used in the PD home diary. Note that subjects without PD also have BKS and DKS, but their medians are lower than PD subjects [7]. For each hourly diary entry, the probability that the BKS or DKS in that hour were higher than 75^th^ percentile was estimated. This was done in the following manner: The 75^th^ percentiles of BKS and DKS of normal subjects were used from data of the study by Griffith and colleagues [7] and the probability that more BKS or DKS was greater than the 75^th^ percentile of controls in each hour was estimated using the Chi^2^ test. There are 30 two minute epochs in an hour and in normal subjects, 1 in 4 will be over the 75^th^ percentile of controls. Thus in patients, if 15 or more BKS or DKS are greater than 75^th^ percentile of controls in the hour then the χ^2^ test provides a p<0.05. This was treated as the objective assessment of “Off” or “dyskinesia”, respectively. These data are referred to herein as raw data.

All hour-time-periods between 6 am and 10 pm were analyzed for the three motor categories (motor Off, On or On with dyskinesia) states, but hour-time-periods were excluded from analysis if there was no response (sleeping time or PKG logger not on wrist for 30 or more minutes in the hour) or if the patient in the diary or the PKG recorded more than one of the three motor categories for that hour. Analysis of PKG data was performed by raters without contact to patients and blinded for diary entries (LC, MH). Only diary entries of the first day of recordings were conveyed to the PKG analyzers for calibration (see below).

### Calibration of PKG data

As well as correlating scores with the consistent threshold (the 75^th^ percentile), we also attempted to model the thresholds used by the patient and thus obtain better correlations. To achieve this, raw PKG data were tuned to model the first day of diary entries by each patient (referred herein as calibrated PKG data) so as to establish their individual thresholds for Off and dyskinetic state. Calibration from that first day was then applied to the subsequent 5 days, with PKG analyzers blinded to diary entries. Calibration was achieved by modifying two parameters: The first was altering the threshold (i.e. the percentile) for achieving the Off state or On state with dyskinesia and the second entailed shifting the hour in which the PKG was analyzed forwards (+) or backwards (-) relative to the diary. Take for example the hour between 9:00–10:00. If the patient appeared to be switching between Off and On at threshold that best correlated with the 65^th^ percentile then this threshold was used for this patient, instead of the 75^th^ percentile. As well, the diary should have been filled in at 10:00 to reflect an assessment of motor state of the whole of the previous hour. If the PKG data in the period 9:30–10:30 was found to correlate better with the diary entry then the PKG was moved forward in time (+ 30 mins) compared to the diary. In each patient time shift and change in threshold was then applied to all subsequent days.

### Statistical analyses

Statistical comparisons of data were calculated using χ2, McNemar test, Fisher’s exact test, Wilcoxon test, or paired t-test as appropriate. Percentage agreement and Cohen’s κ were used to determine inter-rating agreement between assessment methods on the single-hour-level, Pearson’s correlation test was used for correlations on the total-hours-per-day-level (κ or Pearson’s correlation coefficient |r|<0.3 was considered a weak, κ/|r| = 0.3–0.59 a moderate, κ/|r|≥0.6 a strong agreement/correlation). Multiple linear regression modeling was used to test predictive value of depression and diary data for the PKG results as the dependent variable. To estimate the median difference between PKG values for the relevant hour (in calibrated data) when the diary scored the hour as On compared to when the diary was scored as Off (dyskinetic in the case of DKS), a clustered median regression analysis with the binary diary record (“On”/”Off”) for the hour as an input and the PKG values for that hour as an output, with hour-specific values clustered within the patients was used. In addition, to estimate the between-subject variability as the proportion of total variability in diary entries, a random-effect multi-level logistic regression model with PKG score at a given point of time as an input, the respective diary entry as an output, and the patient as the random effect level variable was developed. The proportion of between-subject variability was estimated using the Intra-class Correlation Coefficient (ICC). Since this is a pilot study and no data on PKG movement assessment were available at the time of the study start, we were not able to perform a sample-size calculation and thus aimed for 100 suitable full-day datasets from 20 patients. Pairwise deletion was applied to missing data. Data were analyzed using SPSS 21.0 (SPSS Inc., Chicago, IL) and Stata IC 13 (StataCorp, College Station, TX). If not mentioned otherwise, all data are displayed as means±SD [range] or numbers (%), significance level was set at *P*<0.05 (two-tailed test).

## Results

The total number of subjects was 24 (16 women (67%); age: 65.0±7.4 [54–82] years; disease duration: 11.7±4.3 [2–20] years; Hoehn-Yahr stage in On: 2.1±0.6 [1.0–3] and Off: 2.8±0.9 [1.0–5]; *P< 0*.*001* [Wilcoxon test]; UPDRSIII motor score in On: 13.5±5.5 [5–28] and Off: 26.9±8.8 [10–47]; *P*<0.001 [paired *t*-test]; levodopa equivalent dose per day [25]: 1,106±358 mg [410–1,690 mg]; see S1 Table for further information). All patients displayed at least two types of motor complications, median number of motor complication was 4 (10^th^/90^th^ percentile: 1/6). S2 Table summarizes the various types of motor complications. Mean BDI score was 9.7±6.7 (0–27) and 8 patients showed scores above the threshold for detecting depression in PD (score > 12) [26].

### Correlations of diary and PKG data on the total-hours-per-day level

**Fig 1:**
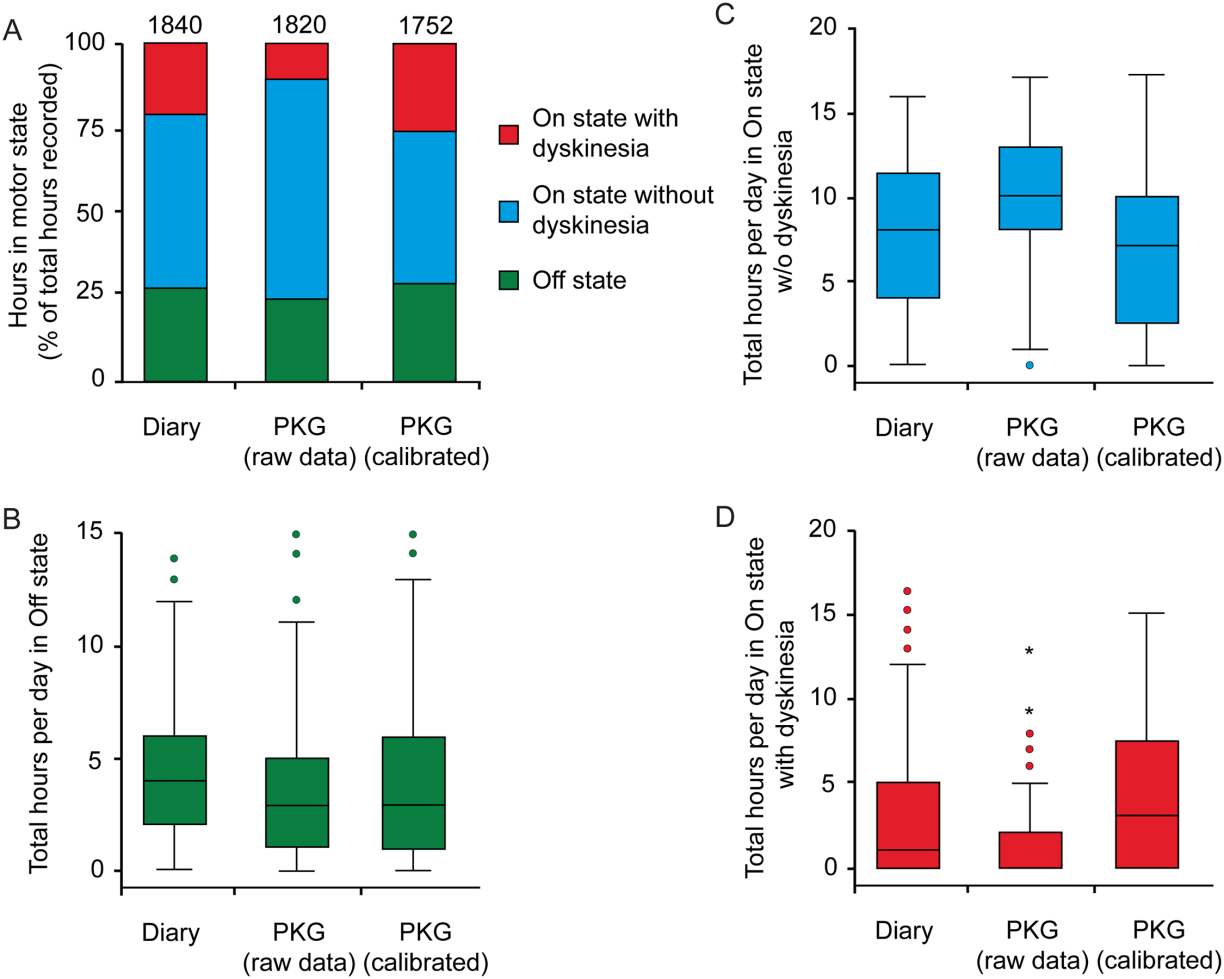
Frequencies of motor states from diaries, raw PKG and calibrated PKG data. **(A)** Distributions of motor states from diaries, raw PKG data and calibrated PKG data recorded over 5 days in 24 patients (120 days). Numbers above bars are total hours available (6 am to 10 pm without sleeping/PKG off time and hours with unclassified motor states). **(B-D)** Displayed are total hours per day in motor Off state (B), motor On state without dyskinesia (C) and dyskinetic state (D) as per the diaries, PKG raw and calibrated data.

From a total of 2,040 hours between 6 am and 10 pm (120 days), 1,840 hour-time-periods for were available for further analysis from diary entries, 1,820 from raw PKG data and 1,752 from calibrated PKG (see S3 Table for details). Fig 1A shows the distributions of motor states for all hours recorded over 5 days in 24 patients with respect to the assessment method (analysis on group level). Distribution of total hours per day in all motor states measured by PKG (calibrated data) closely reflected those assessed by the PD home diaries (Fig 1B–1D). On the patient level, similar results were obtained for the mean number of hours per day per patient collected during the five days from each patient as the common analysis strategy for PD home diary data (S1 Fig). Consistently, we found a moderate correlation between calibrated PKG and diary data for total daily hours in Off and On state without dyskinesia and a strong correlation for the dyskinetic state on the group level (Table 1). The number of motor state switches per day was also similar between techniques with a median of 4 switches per day (10^th^/90^th^ percentile: 1/8) from diaries, 5 switches (1/8) from raw PKG data and 5 switches (2/8) from calibrated PKG data, respectively. Correlations between PKG and diary data for motor state switches per day were generally weak with Pearson’s coefficients between 0.010 and 0.209 (Table 1). Ancillary analyses using multiple linear regression models with depression (as measured by BDI assessment)[26] as an additional independent variable revealed that PKG data were not significantly determined by depression (p>0.05), but prediction of PKG data by diary data remained unchanged for all analyses. Similar results were obtained by the same analysis on the patient level (S4 Table).

**Table 1:** Comparisons of diary data with PKG results.

	Agreement between diary and raw PKG data			Agreement between diary and calibrated PKG data		
	per hour (n, %)	per hour (Cohen’s κ)	per day (Pearson’s r)^c^	per hour (n, %)	per hour (Cohen’s κ)	per day (Pearson’s r)^c^
Motor states pattern	779 / 1,648 (47.3%)^a^	0.080	-	850 / 1,594 (53.3%)^a^	0.304	-
Off state (bradykinesia)	1,067 / 1,648 (64.7%)^a^	0.057	-0.049	1,108 / 1,594 (69.5%)^a^	0.216	0.404***
On state w/o dyskinesia	872 / 1,648 (52.9%)^a^	0.043	0.063	997 / 1,594 (62.5%)^a^	0.257	0.562***
On state with dyskinesia	1,267 / 1,648 (76.9%)^a^	0.176	0.437***	1,188 / 1,594 (74.5%)^a^	0.329	0.658***
Total motor state changes	697 / 1,427 (48.8%)^a^	0.023	-	636 / 1,328 (47.9%)^a^	0.033	-
Motor state switches only^b^	41 / 161 (25.5%)^a^	0.075	0.010	44 / 156 (28.2%)^a^	0.122	0.209*

* indicates p<0.05

*** represents p<0.001

^a^Data are from 2×2 contingency tables analyzing hours/switches with agreement between diary and PKG data versus total hours/switches recorded (e.g. for Off state analysis, there was agreement between diary and raw PKG data (diary-Off/PKG-Off or diary-nonOff/PKG-nonOff) in1,067 hours out of a total of 1,648 hours recording time)

^b^Data are for motor state switches only, excluding hour-to-hour data showing no motor state change; n = 1194 for raw PKG data analysis, n = 1122 for calibrated PKG data analysis)

^c^Displayed data are Pearson’s correlation coefficients r.

**Fig 2:**
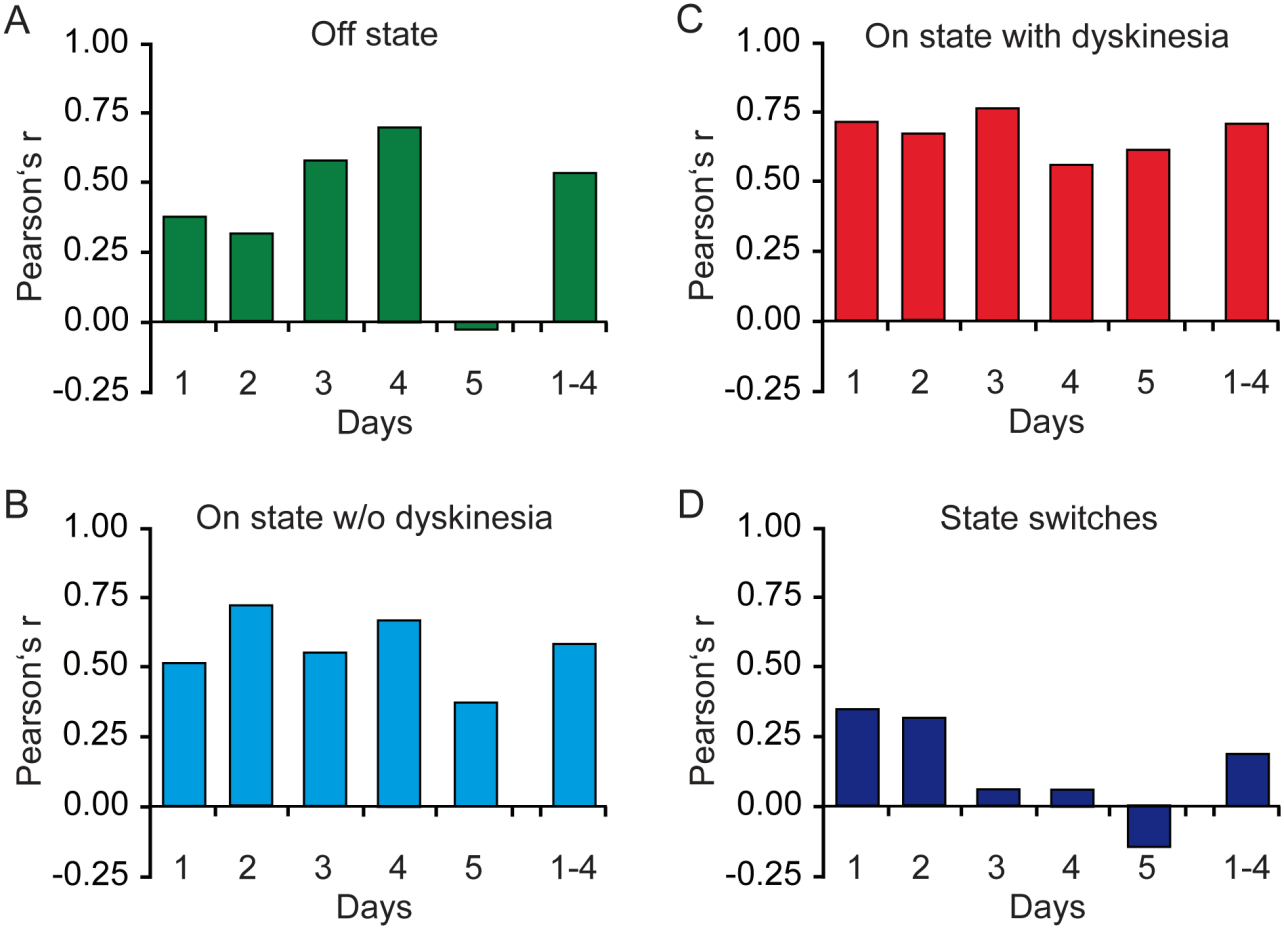
Correlations on the total-hours-per-day-level of diary data with calibrated PKG data with respect to the 5 consecutive days of recording. **A-D)** Displayed are Pearson’s correlation coefficients for correlations on the total-hours-per-day-level for each of the 5 consecutive days of recordings and for the days 1 to 4 for motor Off state **(A)**, motor On state without dyskinesia **(B)**, dyskinetic state **(C)** and for motor state switches **(D)**.

We then analyzed the correlations on the total-hours-per-day level of diary data with calibrated PKG data with respect to the 5 consecutive days of recording (Fig 2). In general, we detected stable Pearson’s correlation coefficients for the various motor states over the 5 consecutive days except for the motor off state with a drop in correlation strength at day 5. Interestingly, we observed a continuous decrease of the correlation strength over time for the number of motor state switches with no relevant correlations for days 3–5 (Fig 2D). Pearson’s correlation tests using the data of only the first 3 or 4 days revealed strong correlation of diary data and calibrated PKG data for all motor states (Pearson’s correlation coefficients r>0.6).

### Correlations of diary and PKG data on the single-hour level

To determine inter-rating agreement as to the state at a particular hour of the day the raw agreement rates (in percent) and Cohen’s κ test for categorical items in 2×2 contingency tables were used. Raw agreement rates were 53 to 77%, but only weak agreement according to the Cohen’s κ scores of 0.043 to 0.176 between diary entries and raw PKG data for motor states and 49% and Cohen’s κ of 0.023 for motor state switches (Table 1). Calibration of PKG data to diary entries on day one led to slightly higher agreement rates of 63–75% with weak to moderate agreements (Table 1). We did not find relevant differences in agreement rates (Cohen’s κ) between days or between hours during the day, except for motor state switches with decreasing agreement rates over time similar as observed at the total-hours-per-day level. In addition, we found differences for motor states as well as motor state switches neither in the agreement rates (p>0.05; Fisher’s exact tests) nor Cohen’s κ values between depressed and non-depressed subjects (Mann-Whitney U-test).

### Factors that influenced calibration

**Fig 3:**
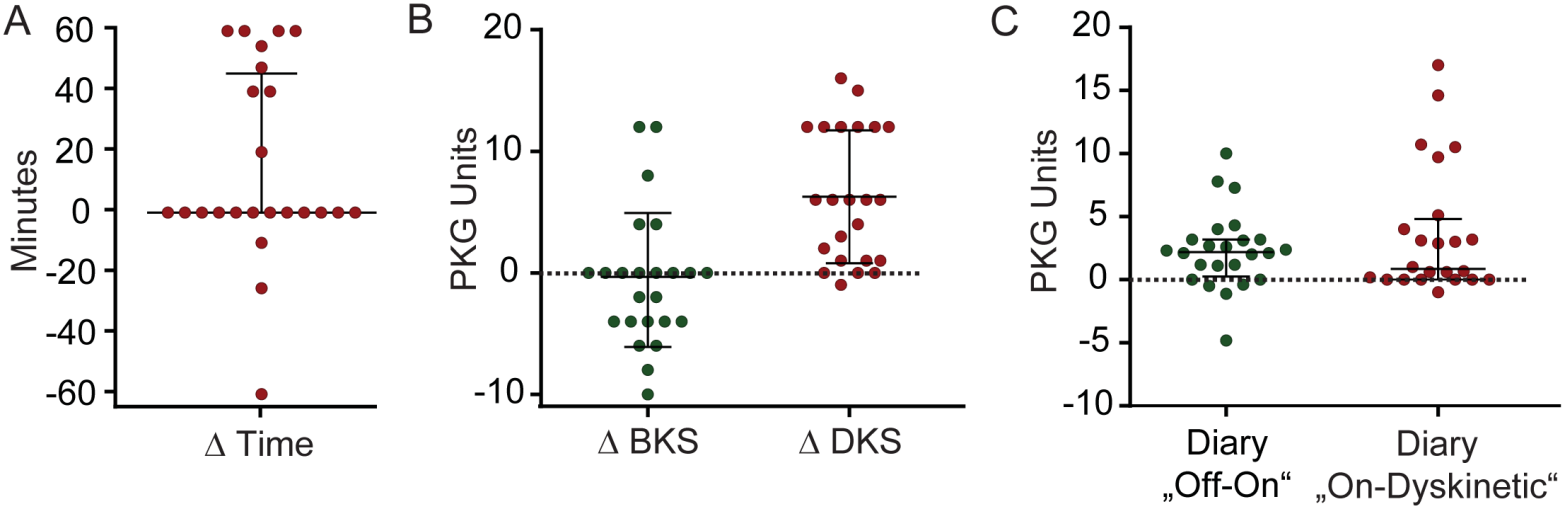
Ancillary analyses of factors that influenced calibration. **A)** Number of minutes that diaries were shifted forwards (+) or backwards (-) relative to the PKG time (see text): 12/24 required no shift in time and 9/12 of the remainder required shifting the PKG forward in time. **B)** Extent to which the threshold for “Off” was increased or decreased from 26 BKS units and 4 DKS units. Note that a reduction in 8 BKS units brings the threshold to the mean of normal subjects. It was necessary to increase the DKS threshold in most subjects. **C)** Value of the regression coefficient (Y axis) which is the difference in the median BKS (green symbols) or median DKS (red symbols) when the diary has been scored as On compared to when the diary scores were Off or dyskinetic (respectively). The bars show the median and interquartile range (IQR).

Calibration of the PKG data was achieved by modifying two parameters: The first was altering the threshold for achieving the Off or dyskinetic state and the second entailed shifting the hour in which the PKG was analyzed forwards (+) or backwards (-) relative to the diary (Fig 3A). Calibration of the timing was not needed in 12/24 subjects, but did require a shifting forward of the PKG relative to the diary in 9/12 subjects. Fig 3B shows the extent to which the threshold for the Off state was increased or decreased from 26 BKS units and for the dyskinetic state changed from 4 DKS units (50^th^ percentiles of normal subjects). In approximately half the patients, the BKS threshold was not changed and in the other cases, the BKS threshold was usually reduced (i.e. less bradykinetic). Note that a reduction in 8 BKS units brings the threshold to the mean of normal subjects. In the case of DKS, almost all subjects required an increase in threshold (19/24 did not consider themselves to be dyskinetic when objective measures would have).

Based on the median regression analysis, the median within-patient difference between the BKS values in hours when the diary is “On” and when the diary is “Off” is 2.37 (95%CI: 0.49–4.25; *P* = 0.014), while the similar difference for DKS (“On” vs dyskinetic hours) is 5.24 (95%CI: 1.18–9.3; *P* = 0.012), illustrated in (Fig 3C). The values of median within-patient differences range from -4.78 to 10 for BKS values and from -1.01 to 14.57 for DKS. Thus about half of the patients required a change in BKS or DKS of >4 to identify the respective motor state (approximates a UPDRSIII >4 and mAIMS >3) [7]. The median differences correlated modestly with the number of changes in motor state (r^2^ = 0.3), suggesting that those who were non-fluctuators were able to detect a smaller change in bradykinesia or dyskinesia to detect the Off or On with dyskinesia state. Random-effect multi-level logistic regression analysis demonstrated that when investigating the association between diaries and bradykinesia within-patient variability is stronger (ICC = 16%) whereas the between-patient differences dominated dyskinesia (ICC = 78%).

## Discussion

Our study revealed moderate-to-high concordance of quantitative motor state assessment using the Parkinson's kinetigraph (PKG) with patient’s diary data when analyzed on a daily basis (total hours per day), but limited concordance on the single-hour-level. In general, raw PKG data showed weak agreement rates, but calibration of PKG data to diary entries from an index day led to higher correlations of PKG data with diary entries on both the daily level and higher inter-rating scale agreement on the single-hour-level. There was a strong correlation of total daily hours in the dyskinetic state with a correlation coefficient >0.6.

Comparing objective measurement devices to subjective assessments such as patient diaries implicates several limitations which might be—at least partially—responsible for their limited agreement. Firstly, the PKG measures movements continuously and classifies them into the motor states for every hour, while the hourly diary completion not only leads to large recall bias, but potentially also due to diary fatigue [4, 5]. However, the amount of incomplete diary entries was similar to the missing number of classifications by the PKG, but diary fatigue might also lead to limited diary entry accuracy fatigue [4, 5]. The assumption of diary fatigue is supported by decreasing agreement rates over days in the present study, but notably we did not directly assess diary fatigue. Secondly, PKG outcomes correlate well with clinical ratings by physicians such as UPDRS or mAIMS suggesting a more objective rating of motor performance, while diary completion is potentially confounded by non-motor symptoms or their fluctuations, particularly psychiatric conditions such as cognitive dysfunction, fatigue and depression. However, persistent depression measured by the BDI was not a relevant confounder for the correlations of diary and PKG data. Whether or not mood or other non-motor symptom fluctuations that are known to appear in conjunction with motor oscillations fluctuations [27, 28] influence the diary ratings needs to be addressed in future studies. Another limitation of the present study is that ratings derived from PKG and patient diaries were not compared to clinical assessments performed by trained clinicians. This would have required continuous presence of a rater during the observation period, which was not part of the protocol. Maybe simultaneous video monitoring would be a way to acquire this supplementary information in future studies. We finally point to the fact that the limits of the use of patient diary information as a gold standard in clinical studies were not focus of our study.

The strong correlation of PKG measures with objective ratings (UPDRS and mAIMS) using the same recording paradigms as in the recent study [7] also implies that the fact that the PKG was placed on one limb only (wrist on most affected side) is not an important reason for a lack of correlation. In addition, PKG might not be able to reliably distinguish between volitional rest and immobility in Off state. In order to minimize these factors, only inpatients under continuous surveillance were enrolled and trained rigorously in completing the diaries correctly. Therefore, the PKG should be combined with a patient activity protocol similar to Holter ECG monitoring.

Another important consideration in this comparison potentially limiting the agreement between diary and PKG data is that the clinical scales and the PKG scores are continuous whereas diaries typically have either a three point scale (Off, On or dyskinetic). Ideally, there should be a specific point on the continuous scale and when this point is exceeded the subject is deemed “Off” or dyskinetic. However with diaries each patient selects their own (possibly varying) threshold. Thus a comparison between the PKG (and any other objective continuous rating scale) with diary entries will require that first some threshold level is chosen and second that an attempt is made to establish what idiosyncratic threshold each subject has chosen. To further add more variability to the patient ratings, the finding that the within-patient variability is stronger for the association between diaries and PKG for bradykinesia, whereas the between-patient differences dominated dyskinesia, might be interpreted as meaning that individual patients are more variable in the way they score Off and On, whereas with dyskinesia, the patients are intrinsically more consistent in their scoring. These factors which were partially eliminated by data calibration during data processing leading to much better agreement rates between PKG and diary data. Indeed, a closer look at the ratings of the different motor states conveys that the calibrated PKG data agree with the diaries classification of the Off state and dyskinetic states for a high proportion of hours (see S5 and S6 Tables). However, it also provides an explanation for why the Cohen’s κ would have been low in many of these patients: That reason is that for many patients, while there was high agreement, it was for being in the On state, with a very small proportion in the bradykinetic or dyskinetic state. Cohen’s κ test and other categorical tests to do not handle this type unbalanced distribution of values in cells.

Calibration of the PKG data consisted of either shifting the time or the threshold. In about a third of subjects, there was a forward shift, meaning that events happening in the PKG at the time of filling in the diary led to better correlations than those of an hour earlier. One explanation might be that the patient’s current state was likely to be more influential than the memory of the last hour. On the other hand, almost half the patients (11 out of 24) required a lowering of the bradykinesia threshold. The objective threshold for the PKG was set at 8 BKS units above “normal”, which is roughly equivalent to a UPDRSIII score of around 10 [7]. This implies that for half the patients this threshold is too high and that they are detecting lower thresholds. The regression analysis showed that about half of the patients required a change in BKS >4 (UPDRS III ~4) to qualify for a change from On to Off state. In the case of dyskinesia, an increase in threshold was required in 19 out of the 24 subjects. The PKG threshold was set at 4 DKS, which is equivalent to an AIMS = 0. If the threshold had been set at the 75^th^ percentile of controls (equivalent to an AIMS ~10) [7], then most subjects would have required a lowering of threshold. The random effect logistic regression model implies that between-patient differences dominated the variability between PKG and diaries in scoring dyskinesia. This may indicate that individual patients are intrinsically more consistent in their scoring of being dyskinetic than in their scoring of bradykinesia.

The improvement of correlations between PKG and diary data by calibrating the PKG data as well as the discussed differences of the correlation strengths between diary and PKG data for motor fluctuations and dyskinesia might not only support the notion that low correlation levels were mediated by individual differences or thresholds in diary entries by the patients, but might also point to potential limits in the algorithm used to classify continuous PKG data into motor states based on upper extremity motor function. The finding that the median BKS and median DKS closely correlate with the UPDRS III and mAIMS support the contention that the original algorithms can classify motor symptoms as rated by an objective observer [7] and can distinguish between fluctuating and non-fluctuating patients [19]. Thus the mismatch seems more likely to be between the objective and subjective observer. However, future threshold-free and adaptive technologies might be utilized to improve categorization of continuous PKG data into categorical diary data and/or other patient ratings.

Together, the PKG is a valuable tool to measure total motor state hours per day particularly for the dyskinetic motor state. Future studies in larger patient cohorts—at best under controlled prospective conditions—are warranted to confirm our data and to evidence the PKG as a suitable trial endpoint.

## Supporting Information

S1 Fig Frequencies of motor states from diaries, raw PKG and calibrated PKG data per day on the patient level. Displayed are the mean number of hours per day per patient collected during the five days from each patient (n = 24) in motor Off state (A), motor On state without dyskinesia (B) and dyskinetic state (C) as per the diaries, PKG raw and calibrated data.
(TIF)

S1 Table Demographic and clinical data. (DOCX)

S2 Table Types of motor complications. (DOCX)

S3 Table Data of PKG recordings and PD home diary entries. (DOCX)

S4 Table Correlation of diary data with PKG results (per day on the patient level). (DOCX)

S5 Table Matching between calibrated PKG and diary data in identification of bradykinesia. (DOCX)

S6 Table Matching between calibrated PKG and diary data in identification of dyskinesia. (DOCX)
