# Supplementary figures and images for: Correlation of Quantitative Motor State Assessment Using a Kinetograph and Patient Diaries in Advanced PD: Data from an Observational Study
Source: PLoS One. 2016 Aug 24;11(8):e0161559. doi: 10.1371/journal.pone.0161559 (PMC4996447; doi:10.1371/journal.pone.0161559)

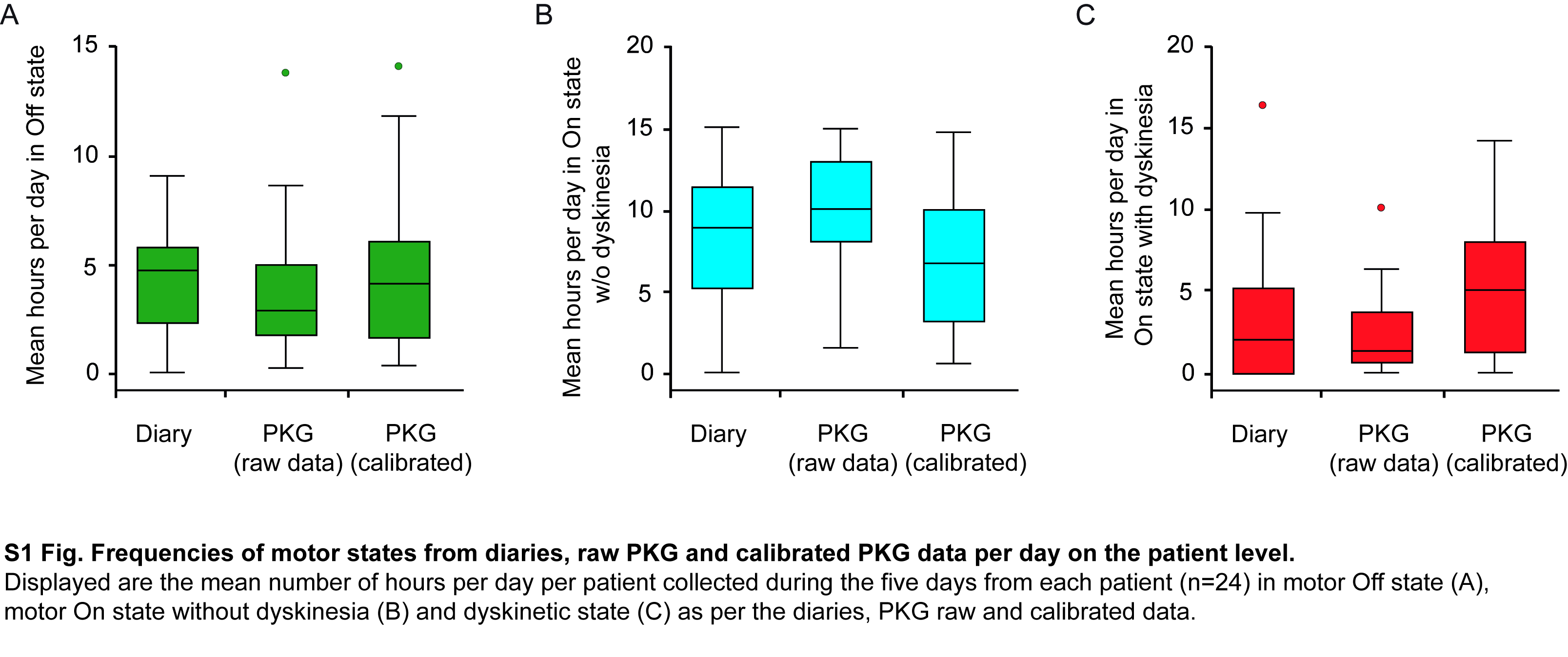

Supplement: S1 Fig — Displayed are the mean number of hours per day per patient collected during the five days from each patient (n = 24) in motor Off state (A), motor On state without dyskinesia (B) and dyskinetic state (C) as per the diaries, PKG raw and calibrated data. (TIF) [file pone.0161559.s001.tif]
