# Supplementary material for: Correlation of Quantitative Motor State Assessment Using a Kinetograph and Patient Diaries in Advanced PD: Data from an Observational Study
Source: PLoS One. 2016 Aug 24;11(8):e0161559. doi: 10.1371/journal.pone.0161559 (PMC4996447; doi:10.1371/journal.pone.0161559)
Supplement: S1 Table — (DOCX) [file pone.0161559.s002.docx]

**S1 Table.** Demographic and clinical data

|  | **Overall cohort** | |  |
| --- | --- | --- | --- |
|  | **On state** | **Off state** | ***P* value** |
| No. of patients | 24 | |  |
| Men / Women | 8 (33%) / 16 (67%) | |  |
| Age (years) | 65.0±7.4 (54-82) | |  |
| Disease duration (years) | 11.7±4.3 (2-20) | |  |
| Duration of motor complications (months) | 46±43 (1-153) | |  |
| Hoehn & Yahr stage | 2.1±0.6 (1.0-3) | 2.8±0.9 (1.0-5) | < 0.001**^◊^** |
| 1 | 3 (13%) | 1 (4%) |  |
| 1.5 | 1 (4%) | 4 (17%) |  |
| 2 | 12 (50%) | 10 (42%) |  |
| 2.5 | 4 (17%) | 4 (17%) | 0.230* |
| 3 | 4 (17%) | 1 (13%) |  |
| 4 | 0 (0%) | 2 (8%) |  |
| 5 | 0 (0%) | 2 (8%) |  |
| PD treatment |  |  |  |
| Levodopa equivalent dose (mg per day) | 1106±358 (410-1690) | |  |
| Levodopa | 23 (96%) | |  |
| Dopamine agonists | 21 (88%) | |  |
| Amantadine | 11 (46%) | |  |
| STN-DBS | 2 (13%) | |  |
| COMT inhibitor  MAO-B inhibitor | 16 (67%)  7 (29%) | |  |
| UPDRS score |  |  |  |
| UPDRS part I score | 2.0±1.7 (0-8) | |  |
| UPDRS part II score | 10.5±5.0 (0-22) | |  |
| UPDRS part III score | 13.5±5.5 (5-28) | 26.9±8.8 (10-47) | < 0.001^#^ |
| ΔUPDRSIII_On/Off_ | -13.4±8.7 (-35 to 0) | |  |
| UPDRS part IV score | 7.8±3.9 (1-14) | |  |
| Schwab & England ADL score | 85.0±8.3 (70-100) | 65.2±16.5 (30-90) | < 0.001**^◊^** |
| BDI score | 9.7±6.7 (0-12) | |  |

Data are mean (SD), median (range), or number (%). Levodopa equivalent doses were calculated according to Tomlinson et al. (2010).

*McNemar-Bowker symmetry test of all Hoehn & Yahr stages between On and Off; ◊Wilcoxon test; # Paired *t*-test.

BDI, Beck’s Depression Inventory; COMT, catechol-*O*-methyl transferase; MAO-B, monoamine oxidase B; STN-DBS, deep brain stimulation of the *subthalamic nucleus*; UPDRS, Unified PD Rating Scale.
