# Supplementary material for: Correlation of Quantitative Motor State Assessment Using a Kinetograph and Patient Diaries in Advanced PD: Data from an Observational Study
Source: PLoS One. 2016 Aug 24;11(8):e0161559. doi: 10.1371/journal.pone.0161559 (PMC4996447; doi:10.1371/journal.pone.0161559)
Supplement: S2 Table — (DOCX) [file pone.0161559.s003.docx]

**S2 Table.** Types of motor complications

| **Motor complication** | **No. of subjects (%)** |
| --- | --- |
| End-of dose akinesia | 14 (58%) |
| On-Off phenomenon | 20 (83%) |
| No On response | 6 (25%) |
| Off period dystonia | 14 (58%) |
| Peak-dose dyskinesia | 14 (58%) |
| Biphasic dyskinesia | 9 (38%) |
| Freezing | 9 (38%) |
