# Supplementary material for: Correlation of Quantitative Motor State Assessment Using a Kinetograph and Patient Diaries in Advanced PD: Data from an Observational Study
Source: PLoS One. 2016 Aug 24;11(8):e0161559. doi: 10.1371/journal.pone.0161559 (PMC4996447; doi:10.1371/journal.pone.0161559)
Supplement: S3 Table — (DOCX) [file pone.0161559.s004.docx]

**S3 Table.** Data of PKG recordings and PD home diary entries

|  | **PD home diary** | **Raw PKG** | **Calibrated PKG** |
| --- | --- | --- | --- |
| Total number of hours between 6 am and 10 pm (120 days) | 2,040 | | |
| Total recording time between 6 am and 10 pm (hours) | 2,040 | 2,006 | 2,005 |
| Hour time periods for analysis | 1,840 (90.2%) | 1,820 (90.7%) | 1,752 (87.4%) |
| Sleeping time/PKG off (hours) | 153 (7.7%) | 146 (7.3%) | 150 (7.5%) |
| Incomplete entries / no classification of motor state by PKG (hours) | 47 (2.3%) | 40 (2.0%) | 103 (5.1%) |
| Total possible motor state changes from hour to hour | 1,775 | 1,608 | 1,494 |
| Motor state switches from hour to hour | 526 (29.6%) | 559 (34.7%) | 574 (38.4%) |

Note that PKG was worn between 6 am and 10 pm.
