# Supplementary material for: Correlation of Quantitative Motor State Assessment Using a Kinetograph and Patient Diaries in Advanced PD: Data from an Observational Study
Source: PLoS One. 2016 Aug 24;11(8):e0161559. doi: 10.1371/journal.pone.0161559 (PMC4996447; doi:10.1371/journal.pone.0161559)
Supplement: S4 Table — (DOCX) [file pone.0161559.s005.docx]

**S4 Table.** Correlation of diary data with PKG results (per day on the patient level)

|  | **Pearson’s correlation coefficient r** | |
| --- | --- | --- |
|  | **Raw PKG data** | **Calibrated PKG data** |
| Off state (bradykinesia)^a^ | -0.055 | 0.374* |
| On state w/o dyskinesia^a^ | 0.203 | 0.611** |
| On state with dyskinesia^a^ | 0.365* | 0.717*** |
| Motor state switches^b^ | 0.116 | 0.364* |

* indicates p<0.05, ** represents p>0.01, and *** represents p<0.001

^a^Displayed data are from the mean number of hours per day per patient collected during the five days from each patient (n=24) in motor Off state (bradykinesia), motor On state without dyskinesia and dyskinetic state

^b^Displayed data are from the mean number of motor state switches per day per patient collected during the five days from each patient (n=24), excluding hour-to-hour data showing no motor state change
