# Supplementary material for: Correlation of Quantitative Motor State Assessment Using a Kinetograph and Patient Diaries in Advanced PD: Data from an Observational Study
Source: PLoS One. 2016 Aug 24;11(8):e0161559. doi: 10.1371/journal.pone.0161559 (PMC4996447; doi:10.1371/journal.pone.0161559)
Supplement: S6 Table — (DOCX) [file pone.0161559.s007.docx]

**S6 Table.** Matching between calibrated PKG and diary data in identification of dyskinesia

| **PKG-DK / Diary-DK (%)** | **PKG-DK/ Diary-DK** | **PKG-nonDK / Diary-nonDK** | **PKG-nonDK / Diary-DK** | **PKG-DK/ Diary-nonDK** | **Total hours recorded** | **PKG & Diary agreement rate (%)** | **Cohen's kappa** | **Sensitivity** | **Specificity** |
| --- | --- | --- | --- | --- | --- | --- | --- | --- | --- |
| 0% | 0 | 60 | 0 | 3 | 63 | 95% | 0,000 | - | 95% |
| 0% | 0 | 66 | 0 | 0 | 66 | 100% | - | - | 100% |
| 0% | 0 | 56 | 0 | 1 | 57 | 98% | 0,000 | - | 98% |
| 0% | 0 | 62 | 0 | 0 | 62 | 100% | - | - | 100% |
| 0% | 0 | 72 | 0 | 0 | 72 | 100% | - | - | 100% |
| 0% | 0 | 53 | 1 | 5 | 59 | 90% | -0,029 | 0% | 91% |
| 0% | 0 | 40 | 13 | 5 | 58 | 69% | -0,142 | 0% | 89% |
| 0% | 0 | 72 | 0 | 4 | 76 | 95% | 0,000 | - | 95% |
| 0% | 0 | 77 | 0 | 0 | 77 | 100% | - | - | 100% |
| 3% | 2 | 42 | 4 | 20 | 68 | 65% | 0,005 | 33% | 68% |
| 3% | 2 | 54 | 2 | 8 | 66 | 85% | 0,218 | 50% | 87% |
| 3% | 2 | 50 | 8 | 3 | 63 | 83% | 0,180 | 20% | 94% |
| 4% | 3 | 49 | 3 | 26 | 81 | 64% | 0,055 | 50% | 65% |
| 9% | 6 | 51 | 4 | 6 | 67 | 85% | 0,329 | 60% | 89% |
| 12% | 7 | 10 | 0 | 42 | 59 | 29% | 0,053 | 100% | 19% |
| 12% | 9 | 36 | 14 | 16 | 75 | 60% | 0,082 | 39% | 69% |
| 13% | 8 | 26 | 10 | 17 | 61 | 56% | 0,044 | 44% | 60% |
| 16% | 9 | 14 | 8 | 24 | 55 | 42% | -0,081 | 53% | 37% |
| 21% | 16 | 32 | 9 | 20 | 77 | 62% | 0,229 | 64% | 62% |
| 22% | 15 | 20 | 22 | 10 | 67 | 52% | 0,069 | 41% | 67% |
| 25% | 18 | 16 | 9 | 30 | 73 | 47% | 0,012 | 67% | 35% |
| 30% | 15 | 14 | 6 | 15 | 50 | 58% | 0,169 | 71% | 48% |
| 36% | 24 | 18 | 14 | 10 | 66 | 64% | 0,269 | 63% | 64% |
| 83% | 62 | 0 | 13 | 0 | 75 | 83% | 0,000 | 83% | - |

PKG-DK and PKG-nonDK indicate that the PKG has scored the patient as On with dyskinesia or Off/On w/o dyskinesia. Diary-On and Diary-Off similarly refer to the patients scoring of On with dyskinesia or Off/On w/o dyskinesia through the diary. Each row refers to a single patient score and patients are ranked according to the proportion of scores where both the diary and the PKG scored the patient as dyskinetic (in percent). In the four adjacent cells are the relevant cells for a contingency table for diary and PKG. The 6^th^ column shows the proportion of scores in which the diary and the PKG agreed (in percent). In the 7^th^ column are the Cohen’s κ scores (note that κ scores are very low when the scores in columns #2 or #3 are very small (i.e. most diary and PKG agree that the patient is rarely dyskinetic). The two columns marked Sensitivity and Specificity report the values for the PKG detecting the dyskinetic state compared to the diaries.
